# Trends of Active Learning in Higher Education and Students’ Well-Being: A Literature Review

**DOI:** 10.3389/fpsyg.2022.844236

**Published:** 2022-04-18

**Authors:** Elsa Ribeiro-Silva, Catarina Amorim, José Luis Aparicio-Herguedas, Paula Batista

**Affiliations:** ^1^Faculty of Sport Sciences and Physical Education, University of Coimbra, Coimbra, Portugal; ^2^Research Unit in Sport and Physical Activity (CIDAF), Coimbra, Portugal; ^3^Centre for 20th Century Interdisciplinary Studies (CEIS20), Coimbra, Portugal; ^4^Faculty of Education, Universidad Internacional de La Rioja, La Rioja, Spain; ^5^Faculty of Sport, University of Porto, Porto, Portugal; ^6^Research Centre in Education, Innovation, Intervention in Sport (CIFI2D), Porto, Portugal; ^7^Centre for Research and Intervention in Education (CIIE), Porto, Portugal

**Keywords:** sustainability, active methodologies, university, well-being, review

## Abstract

This literature Review had the purpose of inspecting how the use of active learning methodologies in higher education can impact students’ Well-being. Considering the Heads of State meeting at United Nations Headquarters on September 2015, in which the 2030 Agenda for Sustainable Development was adopted by all United Nations Member states, this literature review is limbered to the time period between September 2015 and September 2021. A Previous research focused on reviews was made to support the conceptual framework. The search was done in two databases - Web of Science main collection and Scopus - by two researchers autonomously, using the following search criteria: “higher education AND active learning AND student AND wellness OR well-being OR wellbeing.” The studies section attended the following inclusion criteria: (i) published in peer-reviewed journals; (ii) empirical studies; (iii) written in English, French, Portuguese or Spanish; (iv) open access full text; (v) Higher education context; and (vi) focused on the topic under study. The search provided 10 articles which were submitted to an inductive thematic analysis attending to the purpose of this review, resulting in two themes: (i) students’ well-being during confinement; (ii) methodological solutions for students’ well-being. Data show that the use of active methodologies, as digital technologies, and the incorporation of some practice as physical activity and volunteering seems to benefit students’ well-being, namely in their academic achievement, physical, emotional, and social life, and empower them to the professional future with multi-competencies. Higher education institutions need to understand the value of active learning methodologies in sustained education and promote them in their practices.

## Introduction

The well-being of students has grown in importance in recent decades, and according to Ecclestone and Hayes (2009), everyone considers that well-being should be emphasized as a component of education. There are different interpretations of well-being, and several educational policies that consider well-being in different ways. Tiberius (2013) highlights five main theories, with well-being considered as either a subjective theory (based on things that are intrinsically good for us) such as hedonism (Bradley, 2015); desire fulfillment (Griffin, 1986) or life satisfaction (Sumner, 1996), or an objective theory (based on things that are instrumentally good for us) such as human nature fulfillment theory (Nussbaum, 2011) or individually driven nature fulfillment theory (Haybron, 2008). Thorburn (2020) taking as a reference this conceptual framework encompassed in two major perspectives of well-being, pointed out that a “middle-path version of well-being (one that coherently merges the intrinsic and the instrumental, the subjective with the objective) could better answer to the necessity of improvements in subject teaching and personal well-being.” Seeing this understanding of well-being, and the importance of citizens’ well-being for societal growth and sustainability, it is crucial that students’ well-being, the future citizens, be included in national and international policies.

Since the 2000s, there has been a strong interest in educating for personal well-being, Thorburn (2020) reported that countries like Australia, England, New Zealand, and Scotland, in different ways, try to incorporate issues related to students’ well-being in their national curricular reforms. As stated by Matthews et al. (2015), well-being is represented in educational policy when schools display the ability to answer to societal concerns for students’ mental, emotional, social, and physical needs. Norway is an example since it believes that, even in difficult economic circumstances, schools can help to make young people’s lives more rewarding and meaningful (Layard and Dun, 2009).

On a global scale, the United Nations resolution titled “Transforming our World: Sustainable Development Agenda 2030” went into effect on January 1, 2016, along with the 17 sustainable development goals. These intended to build, by 2030, a world with equitable and universal access to quality education at all levels, to health care and social protection, where physical, mental and social well-being are assured (United Nations, 2015, p. 17), emphasizing that no one, whether from developed or developing nations, would be left behind. Concerning education, especially goals three and four, pointed to “ensure healthy lives and promote well-being for all at all ages and ensure inclusive and equitable quality education and promote lifelong learning opportunities for all, respectively” (United Nations, 2015, p. 17).

Without foreseeing the global public health crisis that was to come, the General Secretary of the UN reiterated the Agenda in September 2019, appealing to the need for the current decade to be one of action, so that the goals could be met, with education being one of the primary vectors of change (United Nations, 2020). In fact, the pandemic’s accentuated inequality heightened a digital, educational, and social gap (Díez-Gutiérrez and Gajardo-Espinoza, 2020), exacerbating socio-economic disparities and focusing attention on digital exclusion (Tommaso and Soncin, 2021), jeopardizing even more the 2030 Agenda’s goals by forcing many students, already among the most disadvantaged, to drop out of school (World Bank, 2020).

In this pandemic scenario, the relevance of student well-being has increased. Higher education institutions made significant technology expenditures to set up classrooms despite the fact that each university was free to select how to best organize the transition (Bergdahl et al., 2020; Silamut and Petsangsri, 2020). As a result, the route toward the goals of sustainable development, although always significant, has become both fundamental and complex in this new context, with everyone having a role to play.

Universities, which Batista et al. (2021) claims that, in the context of teacher education, their commitment to society is nothing more than mere declarations of intent, see their responsibility increased here, given that they will have to train teachers for a future they do not foresee, but which they know is constantly changing and updating. Despite the limited academic references relating to how well-being may become a successful element of schooling, curricular adjustments have been taking place in several countries, including Australia, England, New Zealand, and Scotland, in order to integrate questions linked to well-being (Thorburn, 2020).

In the ‘90s, higher education teachers had an intuitive grasp of “active learning,” believing that learning is intrinsically dynamic and that students are actively participating when listening to formal lectures in the classroom (Bonwel and Eison, 1991). This kind of thinking shifted. According to the National Survey of Student Engagement and the Australasian Survey of Student Engagement, active learning includes students’ efforts to actively create their knowledge (Brame, 2016). Students must read, write, discuss, solve problems, and engage in higher-order thinking activities such as analysis, synthesis, and assessment. Students should be involved in doing things and thinking about what they are doing, and students’ explorations of their attitudes and values should be emphasized in active learning practices (Bonwel and Eison, 1991; Carr et al., 2015). Nevertheless, the notion of active learning results not only from teaching methodologies that require students to actively participate in the classes’ activities (to build their own knowledge) but involves other methodologies not related with the subject under study and that lead students to leave the walls of the school space. Carr et al. (2015) referring that this broader understanding of active learning, entails not only working with other students on projects during class, giving a presentation, asking questions or contributing to discussions, but also participating in a community-based project as part of a course, working with other students outside of class on assignments, discussing ideas from a course with others outside the class, and tutoring peers. So, active learning requires viewing the learning process as a constructive process that brings individuals from all over the world together. As stated by Misseyanni et al., 2018 (in preface 2018, p. XIX) “we do believe in the capacity of the global community of creative minds and caring individuals to use active learning for the development of a new culture that will lead to more sustainable societies.” The same authors argued that active learning entails adapting our circumstances, personal beliefs, and understandings to a global scale. According to this remark, higher education programs may empower students to have a more humanistic perspective, as well as for the well-being of their pupils. As a result, teaching approaches must be tailored to people and should aid in their integration into society, so that learning may be transferred to the future active lives of students who do not yet know what they want to do (Sebastiani, 2017).

Collaboration among all is the way to answer to the challenges that the world is currently facing, such as environmental preservation, poverty, socially inclusive and just development, smart and sustainable cities, mutual respect, and the generation of new knowledge for providing sustainable solutions to social problems, is the vision for the active learning philosophy that must be implemented (Misseyanni et al., 2018). Learning can always make a difference in this situation, reducing passivity in the face of challenges, mobilizing emotions, and inspiring action.

This issue prompted us to look at how the use of active learning methodologies in higher education might affect students’ well-being, which is the study’s main purpose. To accomplish this, we conducted a literature review focusing on the use of active learning methodologies in higher education, from the approval of the 2030 Sustainable Development Agenda (2015) and today (2021), in order to understand the sensitivity of higher education institutions to the agenda, based on the studies conducted during that time period.

### What We Know From Previous Reviews?

**FIGURE 1:**
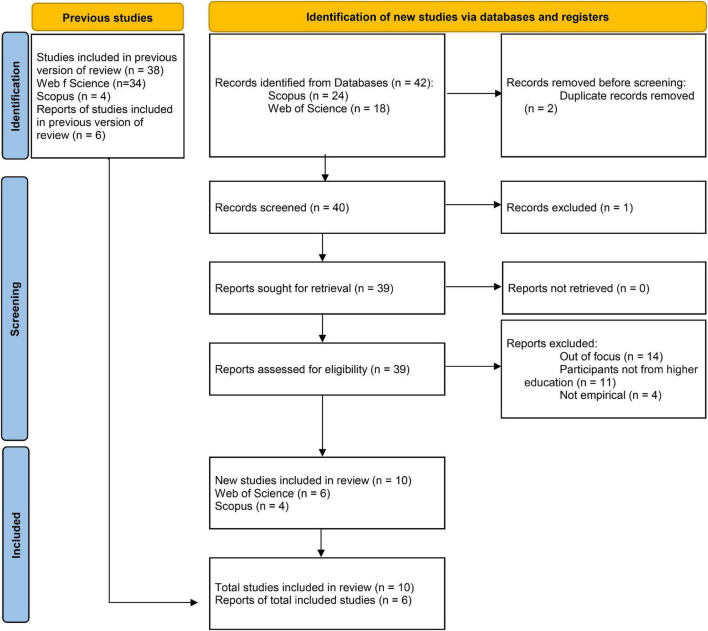
Preferred reporting items for this review.

With the goal of starting from a conceptual framework that would help to frame the focus of the current review, and in response to the component of PRISMA 2020^1^ (previous studies), a search was conducted in the same databases considered for this review (Web of Science and Scopus), in journals that published exclusively reviews or are important in the field of higher education (see Figure 1), resulting on the selection of six studies were (Akinla et al., 2018; van der Zanden et al., 2018; Kötter et al., 2019; Theelen et al., 2019; Thorburn, 2020; Tommaso and Soncin, 2021). These previous reviews approach the issue of student well-being from various angles and with different emphasis; however, the importance of educational procedures focusing on students is a recurring theme.

Some of the arguments in higher education include the stress that the transfer to higher education causes students (Akinla et al., 2018), as well as the desired results of higher education and how such outcomes should be assessed (van der Zanden et al., 2018). By exploring multiple theoretical research strands, the conversation about what it means to be successful at university produced a conceptual framework made of three domains: students’ academic accomplishment, critical thinking skills, and social-emotional well-being. As a result, interventions that promote students’ well-being are critical to their performance. In a research with medical students, Akinla et al. (2018) suggested that near-peer mentorship may help with some of these issues during the transition phase to the university. Near-peer mentoring is a strategy that may enhance students’ professional and personal growth, as well as ease the transition and preserve well-being. In addition to being a significant resource in offering social and academic assistance to incoming students, near-peer mentoring aids in transition and stress reduction. Additionally, customized housing and activity programs (for example, participation in an outdoor orientation program) had a favorable influence on students’ well-being. Multiple research on peer mentorship programs found that involvement aided students’ social integration and adjustment rather than their overall adjustment feelings (van der Zanden et al., 2018).

The pandemic scenario is another issue that makes well-being a crucial concern for higher education. According to Tommaso and Soncin (2021), universities made significant technology expenditures to prepare classrooms for blended learning and strive to provide other activities in addition to teaching. The challenges of the shift from face-to-face to online education were numerous and complex, but they demonstrated that the fundamental goals of the faculties had to be the students, not the method itself. The significance of digital transformations was also emphasized. The difficulty presented has some positive aspects for innovative education. Furthermore, the same authors stated that one of the most essential lessons of this challenge is that the emphasis of education stays on relationships. Relations give meaning to students’ educational experiences as well as the process through which research and innovation are developed (Tommaso and Soncin, 2021).

In a research with preservice teachers, Theelen et al. (2019) reported that computer-based classroom simulations provide a safe tool to practice and develop preservice teachers’ interpersonal competency, as well as contribute to preservice teachers’ well-being. This teacher-student centered strategy aids students in overcoming their difficulties with classroom management and interpersonal relationships between teachers and students. The authors also emphasize the need of investing in simulation active methodologies that improve preservice teachers’ learning experiences and have the potential to be a valuable asset for teacher education by bridging the gap between teacher education and classroom practice. It is necessary to conduct additional research into the interrelationships between preservice teachers’ well-being, interpersonal competency, learning experiences, and computer-based classroom simulations.

According to Thorburn (2020), governmental policies in England, Australia, New Zealand, and Scotland are attempting to interact with well-being goals in education. There is an emphasis on teachers’ agency and students’ overall school-based accomplishments. The intention is to allow teachers to use their professional autonomy to create more comprehensive learning experiences for their pupils.

In a systematic review focused on the protective variables for health and well-being throughout medical education, Kötter et al. (2019) found a considerable variability of potential predictors, with few consistent. However, long-term coping techniques that involve all groups of students as active learners assist them in maintaining their well-being.

Summarizing, despite the various uses and coexistence of different active learning methodologies, the authors are unanimous in recognizing the benefits of student-centered approaches, providing them with experiences that go beyond the class. Learning, according to Sfards’ (1998) conception, needs to be interpreted not only as acquisition, but also as an experience, where learners become active constructors of their learning. Acquiring academic specific knowledge is crucial, however, for that, students need to develop meta-competencies, such as critical thinking, problem-solving ability, and strategies for self-development. In addition, the pandemic situation, with the transition to online teaching, has brought even more emphasis to the need for higher education pedagogy centered on active learning methodologies, in which the teacher educator supports students in the construction of their knowledge and in the social and emotional well-being.

## Materials and Methods

The procedures for this review were guided by Petticrew and Roberts’ (2006) guidelines and the component of the previous studies (present at point 2.1) from of the suggestions of the Preferred Reporting Items for Systematic Reviews and Meta-Analysis (Page et al., 2021) was integrated. The stepwise approach used encompasses (i) formulating search items, (ii) selecting databases, (iii) conducting literature search, (iv) formulating inclusion criteria and applying these criteria to elected relevant literature, and (v) data extraction.

The formulation of search terms and eligibility criteria for searching relevant databases was meant to focus on identifying the most pertinent retrievals related to higher education, well-being, and active learning. The authors believed it was timely to review the interim period from 2015 to 2021, given the milestone that represents the approval in 2015 of the 2030 Agenda for Sustainable Development, in order to understand the sensitivity of higher education institutions to that agenda through the studies developed during that time period. The retrieved studies were considered relevant and included in this review when convincingly connected higher education teaching and student-centered approaches.

### Procedures

#### Databases and Search Terms

Two researchers independently conducted the search in two databases — Web of Science main collection and SCOPUS — using the following search criteria: “higher education AND active learning AND student AND wellness OR well-being OR wellbeing” in all fields. These databases were selected because they contain many of the leading publishers of scientific journals and worldwide databases most relevant to educational research.

#### Eligibility Criteria

The review’s eligibility required empirical studies in open access full text, written in languages understood by the authors (English, Portuguese, French, or Spanish), published in peer-reviewed academic journals, and limited to the period of 2015 to 2021 in open access. The inclusion criteria also required articles that report methodologies, and strategies used in higher education not only to promote university students’ learning but also well-being.

### Selection of Articles and Descriptive Overview

Two of the authors independently conducted the identification of articles provided by the database searches in September 2021 to ensure consensus on relevant articles. The process of screening articles began with a close examination of each of the 42 references (24 from Scopus and 18 from Web of Science) retrieved by the database search (informed by the four specific search terms and the eligibility criteria). Abstracts from the 42 articles were reviewed by the same two authors. Each author independently determined which articles were relevant to the review before comparing data and analyzing any inconsistencies. Both authors agreed that 10 articles were eligible.

From Scopus were excluded 18 articles after analyzing each of them: 11 because they deviated from the focus of this review on the topic itself, five because the participants were not from higher education, and two because they did not present empirical studies in their methodology. This analysis resulted in six final articles included.

As for Web of Science, 14 articles were excluded from the 18 initial articles, of which one was not available, two were duplicated, six due to the participant profile (students not from higher education), three because the theme did not meet the focus for this review, and two for not presenting an empirical study, resulting in four final articles included.

This resulted in a total of 10 articles being considered relevant to the review. The process of selection of references for review and for the previous studies is summarized in Figure 1, according to the three phases (identification, screening and included).

### Data Extraction and Data Analysis

The 10 articles were examined through an inductive thematic analysis (Braun and Clarke, 2012). Four steps were engaged in the theme analysis process: (i) reading each article and noting the main conclusions; (ii) compiling the numerous conclusions of each article in a Word document; (iii) labeling the conclusions of each article with preliminary codes before grouping them into more generic topics; and (iv) organizing the more generic topics into themes. Following the coding of each article’s conclusions, initial codes were iteratively grouped into general subjects and discussed among the authors, culminating in the identification of two themes: (1) students’ well-being during confinement; (2) methodological solutions for students’ well-being.

## Results

Regarding the year of publication, Scopus has three articles from 2021, two from 2020, and one from 2019, whereas Web of Science has one from 2021, two from 2020, and one from 2018. Taking both databases into account, four papers were published in 2021, four in 2020, one in 2019, and one in 2018. All the articles are written in English and any document from 2015 to 2017 fulfills our set of inclusion requirements.

The investigations were conducted in seven countries: three in Spain (Díaz-Iso et al., 2020; Fernández et al., 2020; Luque-Suárez et al., 2021), two in the United Kingdom (Mayew et al., 2020; Defeyter et al., 2021), and one in Germany (Paulus et al., 2021), Taiwan (Dutta et al., 2021), Israel (Martin et al., 2020), the United States of America (Vatovec and Ferrer, 2019), and Sweden (Bälter et al., 2018). Most studies included students in higher education (as it was an inclusion criterion), however, Bälter et al.’s (2018) investigation included also nine teachers. Those students’ (and teachers’) scientific fields are distinct such as medicine (Martin et al., 2020), engineering (Bälter et al., 2018; Paulus et al., 2021), education (Luque-Suárez et al., 2021), human health and the environment (Vatovec and Ferrer, 2019). The remainder studies did not specify the scientific areas of the participants but stated that they belong to various areas, colleges, or universities.

Six of the authors’ approaches were fundamentally quantitative, with questionnaire searching as data collecting instrument (Fernández et al., 2020; Mayew et al., 2020; Defeyter et al., 2021; Dutta et al., 2021; Luque-Suárez et al., 2021; Paulus et al., 2021), and four employed a mixed methods approach, where other instruments were used alongside the questionnaires, such as interviews (Bälter et al., 2018; Díaz-Iso et al., 2020), life experiences (Martin et al., 2020), and intervention reports (Vatovec and Ferrer, 2019). Table 1 displays the results and provides an overview of the research found.

**TABLE 1:** Articles included in the literary review.

Title	Author, Year Country	Method, Instruments, Participants	Objective	Main results
Standing breaks in lectures improve university students’ self-perceived physical, mental, and cognitive condition	Paulus et al., 2021 Germany	Quantitative Questionnaire 582 university students	Examine the impact of extended sitting on students’ self-perceived physical, mental, and cognitive health.	Students’ self-perceived cognitive abilities improve when they spend the break sitting, but their physical and mental well-being suffers. Standing breaks in university lectures are a simple and successful approach to break up students’ sitting time that does not need the presence of a teacher.
Interpreting usability factors predicting sustainable adoption of cloud-based e-learning environment during Covid-19 pandemic	Dutta et al., 2021 Taiwan	Quantitative Structured questionnaires online 256 university students	Investigate the functional relationship between attitudinal readiness, subjective well-being, and cloud-based e-learning adoption intention.	The impact of self-efficacy on adoption intention varies across students who want to utilize it and those who don’t. Analytical elements for attitudinal preparedness, subjective well-being, and adoption intention include a tight interaction between instructors and students, students’ self-governing adaption throughout class, and mutual support and referents among peers. As a result, instructors must pay particular attention to the subtle changes in the instructor-student relationship, students’ psychological and learning conditions.
Mental well-being in United Kingdom higher education during Covid-19: Do students trust universities and the government?	Defeyter et al., 2021 United Kingdom	Quantitative Questionnaire 600 university students	Study of mental well-being and recreancy focuses on the role of universities and government regulators within the education sector.	Confidence in institutions and regulators might play a significant role in students’ mental health during ecological disasters. Students may have grown to rely on university and government organizations to preserve their mental well-being, but now believe these actors can no longer be trusted. Findings suggest universities should pay greater attention to the link between trust and mental health.
Promoting emotional and social well-being and a sense of belonging in adolescents through participation in volunteering	Luque-Suárez et al., 2021 Spain	Quantitative Questionnaire 985 university students	Assess the systematic mechanisms that impact students’ volunteering decisions, as well as the connections between volunteer motivation and the degree pursued.	Volunteering is becoming more connected with good characteristics that aid in the improvement of mental and physical health. Volunteering may be a therapeutic way of dealing with emotions of despair or solitude. It can also enhance self-esteem and people’s lives through encouraging emotional well-being.
The impact of audience response platform mentimeter on the student and staff learning experience	Mayew et al., 2020 United Kingdom	Mixed Questionnaire 204 university students	Providing evidence of effect for lecturers aiming to improve student learning environments while keeping in mind the underlying pedagogy that supports new practices.	Several respondents cited a shift away from passive teaching sessions, a greater emphasis on staff-student and peer-peer conversation in accordance with dialogic teaching approaches, and a more responsive approach to session material. Mentimeter has the potential to increase student pleasure, engagement, voice, and learning, as well as provide a more dynamic and fascinating teaching role for the lecturer.
Evaluation of the emotional and cognitive regulation of young people in a lockdown situation due to the Covid-19 pandemic	Fernández et al., 2020 Spain	Quantitative Questionnaire 1910 responses from more than 80 universities in 13 different Spanish-speaking countries	Examine the pupils’ cognitive-emotional control, as well as their ideas and views concerning the epidemic and the lockdown.	One of the study’s conclusions is the students’ self-evaluation of their digital competence and opportunity for improvement in virtual communicative engagement. University students have to substantially alter their study habits in order to adapt to a new teaching method. Without feeling of optimism, passion, confidence in their digital talents, and social support, none of this would have been possible.
Understanding the role of social interactions in the development of an extracurricular university volunteer activity in a developing country	Díaz-Iso et al., 2020 Spain	Qualitative In-depth interviews 23 university students	Explore whether students believe that participating in structured extracurricular activities has a favorable influence on their academic training, professional growth, university adjustment, psychological well-being.	Students believe that engaging with other students and those at risk of social exclusion might help them improve their academic and professional practices. Findings imply that encouraging volunteer activities in higher education has a variety of benefits. It allows university students to build knowledge shared with others and develop personal and social skills.
Shared living experiences by physicians have a positive impact on mental health attitudes and stigma among medical students: A mixed-method study	Martin et al., 2020 Israel	Mixed Questionnaire and life experiences 53 quantitative study 19 qualitative study Second-year medical students	Determine the effects of physicians sharing their personal stories of overcoming major life problems as an educational intervention to prevent mental health stigma and self-stigma.	When medical culture is constructed on a secret curriculum of stoic perfectionism, trainees may feel as though there is no tolerance for mistakes or sharing personal flows among physicians. Senior physicians sharing personal histories of vulnerability can assist to de-stigmatize mental health and normalize help-seeking among medical students.
Sustainable well-being challenge: A student-centered pedagogical tool linking human well-being to ecological flo urishing	Vatovec and Ferrer, 2019 United States of America	Mixed Intervention reports and questionnaires 35 university students	Determine whether students would uncover positive elements of human conduct that can contribute to both human and ecological well-being.	Students who undertook the Sustainable Well-Being Challenge (SWBC) had a mean rise in positive affect and a mean decrease in negative effect on the Positive and Negative Affect Schedule scale, depending on the activity. Participants were able to recognize the link between their own well-being and the ecological sustainability of each activity.
Walking outdoors during seminars improved perceived seminar quality and sense of well-being among participants	Bälter et al., 2018 Sweden	Mixed Questionnaires and interview 140 participants: 131 university students, 9 teachers	Conduct a feasibility study on how to include physical activity into regular teaching activities for students and teachers, as well as to research how students and teachers viewed the variations in well-being between outdoor walking seminars and normal indoor seminars.	According to both the students and the professors who led the seminars, a sense of well-being may be achieved as the seminars’ perceived quality improves. Incorporating comparable types of outdoor walking into normal work days might provide a number of health and educational benefits. It is insufficient to encourage people to become more physically active.

In view of the results obtained, we can observe that four of the five articles from 2021 (the year with the most publications) focus on the well-being of students, particularly mental and emotional, during their confinement due to COVID-19, as does one of the four articles from 2020. The discussions will be structured according to the two main themes identified: (i) students’ well-being during confinement; and (ii) methodological solutions for students’ well-being.

## Discussion

The study main purpose was to look at how active learning in higher education might affect students’ well-being. This literature review focused on the use of active learning methodologies in higher education in order to understand the sensitivity of higher education institutions to the 2030 Sustainable Development Agenda. The studies included in this review pointed that students’ well-being was an issue under study, considered during confinement period and in relation of diferents active methodologies used by university teachers.

### Students’ Well-Being During Confinement

Fernández et al. (2020) discovered that developing a virtual communicative relationship was a way to reduce emotions of loneliness or social isolation. This similar assumption, about the favorable effects of online classes on student well-being, is evident in a Theelen et al.’s (2019) study with preservice teachers. According to the authors, computer-based classroom simulations offer a safe approach for preservice teachers to practice and develop their interpersonal competency, which contributes to their well-being. This teacher-student centered strategy assists them in overcoming issues with classroom management and teacher-student interpersonal interaction.

Dutta et al. (2021) also looked at how Taiwanese students used digital technologies during confinement, focusing on what they called a “sustainable cloud-base e-learning system,” which defined a learning configuration that included data and communications and allowed for the creation and execution of innovation within an e-learning system. The findings highlight that self-efficacy has a significant impact on students’ predisposition to utilize or not use ‘digital cloud technologies’ functioning as a facilitator of student behavior.

However, as Thorburn (2020) points out, public policies in England, Australia, New Zealand, and Scotland are attempting to interact with well-being goals in education. There is an emphasis on instructors, agency, and students, as well as broader school-based accomplishments. The goal is to provide instructors greater professional autonomy so they may create more comprehensive learning experiences for their pupils. In the same context, Defeyter et al. (2021) attempted to understand why some English students showed low levels of mental well-being during confinement, with the findings indicating that the lack of confidence in the performance of their respective universities and governments in the face of ecological disasters has an impact on their mental well-being, as it transmits a sense of insecurity and uneasiness.

With an identical intention, Luque-Suárez et al. (2021) focused on ways to overcome the mental and emotional distress caused by the first confinement of Spanish students, defending the voluntary work performed by those students as a very positive way to find them again. Promoting emotional well-being improves self-esteem and people’s lives by restoring emotional balance and coping with feelings of depression or isolation. Simultaneously, the shortage of employment, which existed previous to the pandemic but has been exacerbated by it, leads Spanish students to regard volunteering as a method to get into the labor market.

In summary, these four studies looked at the effects of confinement on university students’ mental and emotional well-being, attempting to understand the causes and methods for maintaining well-being (Fernández et al., 2020; Defeyter et al., 2021), confirming the role of digital technologies in their daily lives (Dutta et al., 2021), and compensating for the wear and tear of that period through the development of voluntary work (Luque-Suárez et al., 2021).

Despite their undeniable interest, these studies seem to be discrete pedagogical experiences conducted by groups of researchers and focusing on extremely specific features, rather than strategic and anchored goals of higher education institutions.

Given that the pandemic has increased the risk of public mental health problems (Zhang et al., 2020) and that approximately 30 million university students worldwide have had to transition from traditional learning to virtual learning (Wang and Zhao, 2020; Hermassi et al., 2021), it would be expected to investigate and expand teaching through active learning methodologies in higher education, in which students’ autonomy and decision-making capacity, but also cooperation and experience sharing, are the focus. Learning using these approaches would lessen the sense of malaise caused by isolation (or even loneliness) and ambiguity of the situation, since they transform into a contextualized and self-responsible learning process that takes into account each individual’s skills and restrictions.

### Methodological Approaches for Students’ Well-Being

This is where we find the least recent articles, with just one from 2021, three from 2020, one from 2019, and one from 2018. In general, they are simple research documenting active approaches used in higher education. The main goal is to empower students given their academic accomplishment, critical thinking skills, and social-emotional and contribute to their well-being (Akinla et al., 2018; van der Zanden et al., 2018). In studies with German and Swedish students, Bälter et al. (2018) and Paulus et al. (2021) concluded that a sedentary lifestyle is detrimental to higher education students’ commitments and results, proposing breaks in academic activities with moments of physical activity (Paulus et al., 2021), and holding seminars outside (Bälter et al., 2018), an idea that we had already found in the review study by van der Zanden et al. (2018). These results put in evidence the necessity to include well-being in educational policies in schools. Matthews et al. (2015) defends that society needs to take attention to students’ mental, emotional, social, and physical state.

Martin et al. (2020) found that discussing vulnerable situations that occurred throughout the professional life of experienced doctors with Israeli medical students had a highly good effect on the latter’s well-being, making them realize that there is room for failure. This concept of near-peer mentoring as a means of promoting personal and professional development and social integration of students had already emerged in the review study by van der Zanden et al. (2018), namely in the transition to higher education (Akinla et al., 2018). Near-peer mentoring, in addition to being a valuable resource in providing social and academic support to new students, also helps to facilitate transition and a reduction in stress levels.

The studies by Díaz-Iso et al. (2020), with Spanish students, and Mayew et al. (2020), with English students, focus on well-being through a sense of social integration resulting from the interrelationship promoted by extracurricular volunteering (Díaz-Iso et al., 2020), and from the use of a digital platform. Communication between students becomes (more) inclusive as a result (Mayew et al., 2020) due to the use of that digital platform that has the potential to give a voice to less interventionist pupils for whatever reason, whether gender, culture, disability, or other.

Finally, in a broader ecological context, Vatovec and Ferrer (2019) determined that students’ well-being is directly related to their perceptions of the ecological sustainability of each activity they engage in.

Attempting to link the research based on the methods used or advised to attain well-being, three articles link the feeling of well-being of students with the usage of (new) digital technologies (Fernández et al., 2020; Mayew et al., 2020; Dutta et al., 2021); in two of them, well-being is associated with the practice of physical activity as a way to counteract a sedentary lifestyle, which is considered detrimental to individuals’ well-being (Bälter et al., 2018; Paulus et al., 2021); and in two others, it is associated with the performance of volunteer work (Díaz-Iso et al., 2020; Luque-Suárez et al., 2021). According to Thorburn (2020), this kind of methodologies emphasize not only teacher’s agency but also students’ agency. In this way teachers can use their professional autonomy to create more comprehensive learning experiences for their pupils.

Seeking to recognize active methodological forms in higher education, it appears that those that incorporate some practice of physical activity to balance sedentary life, and those that carry out some volunteer practice greatly favor the well-being of students, whether in their academic, professional, physical, emotional, and social perspectives. Regardless of the approaches identified as possible predictors of student well-being, the consistency is low. Long-term coping techniques that involve all groups of students as active learners assist them in maintaining their well-being.

Even in a time of pandemic and subsequent confinement, it was expected that teaching through active methodologies, which were translated into work proposals that implied communication and cooperation between teachers and students, would have increased in an attempt to alleviate the isolation in which everyone was and controlling the harmful emotional effects. However, while there was some concern for the students’ well-being, it was restricted to pedagogical experiences and/or focused on instrument use.

So, despite these results, and although some countries (and some higher education institutions) are attempting to integrate well-being goals into education, as seen in England, Australia, New Zealand and Scotland (Thorburn, 2020), the global picture is not encouraging, given the 193 United Nations members who have signed the Organization’s 2030 Agenda for Sustainable Development. This was clear when we identified that, since the signing of the 2030 Agenda for Sustainable Development, no study from 2015, 2016, or 2017 was found in the two databases used, and just one in each of the subsequent years (2018 and 2019). Despite the inclusion criteria including French, Spanish and Portuguese, only articles in English were found. This brings us back to Batista et al. (2021), who state that when we try to critically evaluate the work performed by universities in regard to their commitment to society, we find nothing promising beyond simple declarations of intent.

Higher education institution policy is a key impediment to social innovation and preserving ethical consistency between what is claimed and done in vocational training and what is actually done in reality of classrooms in higher education. In this regard, structural changes at both macro (institutional) and micro (educational practice) levels are required to lead to an understanding of the value of active learning methodologies in sustained education, because they are situated, meaningful, and contextualized, making the most of resources with view to multi-competence learning, which forms citizens with rights (Vallaeys, 2021). According to Thorburn (2020), the lack of consistency between national and international policies on teaching for well-being hampers the role of schools and teachers, who are tasked with juggling a plethora of tasks. Simultaneously, they must prepare how to include and respond to the new imperatives of personal well-being policies. In light of this, Thorburn (2020) argues that it is critical to devise a feasible plan for improving students’ progress and allowing teachers to make greater use of their professional autonomy.

## Conclusion

Notwithstanding the modest amount of studies found, the results show that the use of active learning methodologies (in and out of class) in higher education positively impacts students’ well-being, particularly, in their academic accomplishment, physical, emotional, and social lives, and to equip them with multi-competencies for their professional future. Nevertheless, there is some alienation or even lack of interest on this subject from the scientific community and, eventually, from higher education institutions themselves.

Concerning the understanding of higher education institutions to the 2030 Agenda, where universal literacy is aspired through equitable and universal access to quality education at all levels, to health care and social protection, and where physical, mental, and social well-being are assured. However, that sensitivity is still tenuous and interpreted in a very limited and geographically circumscribed way. Despite the Agenda’s *recruitment* of all social sectors, including the scientific and academic community, and its appeal to transparent, effective, and accountable institutions, it appears to us that universities are slow to recognize the significance of their role in this entire process, as well as the ways to play it.

Furthermore, with the massive global public health problem that we have been experiencing since the beginning of 2020, it was expected that the panorama would change significantly. Given the gravity of the situation, we cannot consider the ten articles discovered as a good number, even when the results show that the majority of the objectives of the studies pursue things that are instrumentally good for us, converging to an essentially objective interpretation of well-being. This number is one of the study’s limitations, as is the fact that it only represents seven countries, of which, five from the European Continent, one from North America, and one from Asia. This reveals a lack of sensitivity to the study of student well-being while using active learning methodologies, whatever its interpretation, in socially disadvantaged regions or countries. We believe that investing in research on this topic in teams that mobilize different higher education institutions, or even international scientific networks that may include institutions and researchers from different continents, will be one way to overcome this limitation, allowing not only the geographical expansion of research, but also broader interpretations of well-being.

Finally, given the importance of this topic, we believe that in future studies, the number of research databases should be expanded beyond Scopus and Web of Science, allowing for an increase in the number of articles as well as a greater diversity and representativeness of other countries or regions.

## Author Contributions

All authors listed have made a substantial, direct, and intellectual contribution to the work, and approved it for publication.

## Conflict of Interest

The authors declare that the research was conducted in the absence of any commercial or financial relationships that could be construed as a potential conflict of interest. The reviewer CS-G declared a shared affiliation, with no collaboration, with one of the authors, JA-H to the handling editor at the time of the review.

## Publisher’s Note

All claims expressed in this article are solely those of the authors and do not necessarily represent those of their affiliated organizations, or those of the publisher, the editors and the reviewers. Any product that may be evaluated in this article, or claim that may be made by its manufacturer, is not guaranteed or endorsed by the publisher.

## Footnotes

1 The PRISMA 2020 statement replaces the 2009 statement and includes new reporting guidance that reflects advances in methods to identify, select, appraise, and synthesize studies. The structure and presentation of the Prisma have been modified to facilitate implementation (see Page et al., 2021). Besides the identification of studies in databases, the new PRISMA offers the possibility to include previous review studies and identification of other studies using other tools as websites, organizations, and manual search.
